# Comprehensive Management of Patellofemoral Pain Syndrome in a Recreational Long-Distance Runner: A Case Report

**DOI:** 10.7759/cureus.64706

**Published:** 2024-07-17

**Authors:** Chaitali S Vikhe, Swapnil U Ramteke

**Affiliations:** 1 Department of Sports Physiotherapy, Ravi Nair Physiotherapy College, Datta Meghe Institute of Higher Education and Research, Wardha, IND

**Keywords:** proprioceptive deficits, physiotherapy intervention, multifactorial etiology, recreational runners, sports physiotherapy, patellofemoral pain syndrome

## Abstract

Patellofemoral pain syndrome (PFPS) is a major concern in the field of orthopedic medicine, affecting a substantial portion of the population and significantly impacting the quality of life. This condition, characterized by anterior knee pain increasing with various activities, shows higher susceptibility in certain demographic groups, such as women and adolescents. PFPS arises from a multifactorial etiology involving anatomical, biomechanical, psychological, and social factors, making it a complex condition to manage. Despite numerous therapeutic interventions available, including strengthening exercises, manual therapy, and patellar realignment techniques, the long-term efficacy of these interventions remains debated. This case report describes the case of a 21-year-old female recreational long-distance runner with bilateral knee pain diagnosed with PFPS. Through a comprehensive intervention plan targeting strength, function, flexibility, proprioception, and pain management, significant improvements were observed in pain levels and functional outcomes after physiotherapy intervention. This case underscores the importance of a holistic approach in managing PFPS and highlights the need for further research to optimize treatment strategies and improve patient outcomes.

## Introduction

Patellofemoral pain syndrome (PFPS) is a prevalent and consequential condition affecting a substantial portion of the population, with a prevalence of 22.7% [1]. It stands out among knee disorders accounting for 25-40% of cases and significantly impacting their quality of life (QOL) [2]. PFPS exhibits a higher predisposition in specific demographic groups, particularly women and adolescents without structural or significant pathologic changes in the articular cartilage. This demographic susceptibility underscores the need for targeted interventions and management strategies [3]. The hallmark of PFPS is pain exacerbated by various activities that load the knee joint, such as squatting, running, and stair climbing. These symptoms greatly limit the daily activities and QOL of individuals [4]. It is evident in medical settings that PFPS represents 11-17% of knee pain cases in general practice and a significant 25-40% in sports injury clinics [5].

PFPS is a multifactorial clinical condition stemming from abnormal patellofemoral joint loading, leading to increased joint stress and retropatellar pain [6]. The etiology involves a complex interplay of anatomical and biomechanical factors, making it a challenging condition to treat [7]. The syndrome is often described as a "black hole" in orthopedic medicine due to the absence of a single explanation or therapeutic intervention capable of addressing all aspects of patellofemoral dysfunction [8,9]. Common treatment approaches for PFPS include physiotherapeutic interventions, such as strengthening exercises, manual therapy procedures, and patellar realignment techniques. However, the effectiveness of these interventions remains debated, with limited evidence supporting their long-term efficacy [10,11]. Despite positive short-term outcomes, long-term management of PFPS remains challenging, with a significant proportion of individuals experiencing recurrent or chronic symptoms [12].

Furthermore, individuals with concurrent patellofemoral dislocation may face proprioceptive deficits, implicating damaged neuro-proprioceptive structures [13,14] The aim of this case report is to investigate the multifaceted nature of PFPS and to explore effective strategies for managing recurrent pain and preventing its recurrence, particularly in runners.

## Case presentation

A 21-year-old female recreational long-distance runner presented to the sports physiotherapy department due to experiencing bilateral knee pain, which was significantly impacting her running ability. She reported a six-month history of bilateral retropatellar pain, gradually onset over a month without specific trauma or injury. The pain is exacerbated by activities involving prolonged knee flexion, such as ascending stairs, rising from a chair, prolonged sitting, deep squatting, and patellar maltracking during walking. The patient found that symptoms eased with rest, particularly if she avoided sitting for longer than 30 minutes with her knees in a flexed position. After examination, she was diagnosed with PFPS, a common condition characterized by anterior knee pain exacerbated by activities, such as running, squatting, and stair climbing.

Clinical findings

The patient's informed consent was obtained before the examination, following which a physical assessment was conducted. On assessment, there was a decrease in range of motion, strength, function, and proprioception. The patient reported pain levels using the Numerical Pain Rating Scale (NPRS). For pain while running, the patient reported a level of 7 out of 10, and for pain at rest, the patient reported a level of 3 out of 10. Table 1 illustrates the pre-intervention and post-intervention findings with specific assessment parameters.

**Table 1:** Pre-intervention and post-intervention findings with specific assessment parameters NPRS: Numeric Pain Rating Scale

Assessment	Pre-intervention	Post-intervention
Function	Impaired functional movements (single-leg squats, double-leg squats, and step-down tests)	Improved stability and function
Proprioception	moderately impaired, as assessed using a goniometer at 45 degrees of knee flexion	Improved
Patellar tracking	Lateral patellar tracking observed	Improved alignment
Pain assessment by using the NPRS	Running: 7/10 Stairs: 8/10 (ascending) Stairs: 7/10 (descending) Deep squatting: 9/10	Running: 0/10 Stairs: 2/10 (ascending) Stairs: 1/10 (descending) Deep squatting: 2/10
Flexibility	Tightness in hamstrings iliotibial band (Ober’s test - positive) Hip flexors (Thomas test- positive)	Improved flexibility
Neuromuscular Control and Balance by using Star Excursion Balance Test	Moderate instability	Improved stability and balance

Table 2 presents the results of manual muscle testing conducted on both lower limbs before and after the physiotherapy intervention. 

**Table 2:** Pre- and post-intervention manual muscle testing of both lower limbs

		Pre-intervention		Post-intervention	
Joint	Muscles	Left	Right	Left	Right
Hip	Flexor	4	4	5	5
	Extensor	4	4	5	5
	Abductors	4	4	5	5
	Adductors	4	4	5	5
	Internal rotators	3	3	5	5
	External rotators	3	4	5	5
Knee	Flexor	3	3	5	5
	Extensor	3	3	5	5
Ankle	Dorsiflexors	5	5	5	5
	Planter flexors	5	5	5	5

Pre-intervention

The subject showed a variation in achieving the target angle, with an average deviation of 3 degrees

Post-intervention

The subject's accuracy improved significantly, with an average deviation of 0 degrees from the target angle, as shown in Table 3.

**Table 3:** Knee joint positional sense by using a goniometer

Phase	Trial 1 (degrees)	Difference (degrees)	Trial 2 (degrees)	Difference (degrees)	Trial 3 (degrees)	Difference (degrees)
Pre-intervention	49°	+4°	43°	-2°	48°	+3°
Post-intervention	45°	0°	46°	+1°	44°	-1°

Physiotherapy intervention 

Table 4 outlines the specific interventions implemented during the physiotherapy treatment for PFPS [15].

**Table 4:** Physiotherapy intervention for patellofemoral pain syndrome (PFPS)

Sr no.	Goals	Intervention	Dosage	Frequency
1	To Improve strength	Strengthening of hip adductors and lateral rotators	10 reps × 3 sets	Thrice weekly for four weeks
2	To Improve function	Closed kinetic chain exercises for knee - terminal knee extension with a theraband - wall squats - step-up	30 s hold with three repetitions with progression	For four weeks with progression
3	To Increase flexibility	Stretching: hamstring muscle, gastrocsoleus, and Iliotibial band	30 s hold with 10 reps, three sets	Thrice weekly for four weeks
4	To enhance neuromuscular control and joint awareness	Proprioceptive exercises using a wobble board	10 minutes	Thrice weekly for four weeks
5	To Reduce pain and Improve patellar alignment	Mcconnell patellar taping	1 session	1 session/week

**Figure 1:**
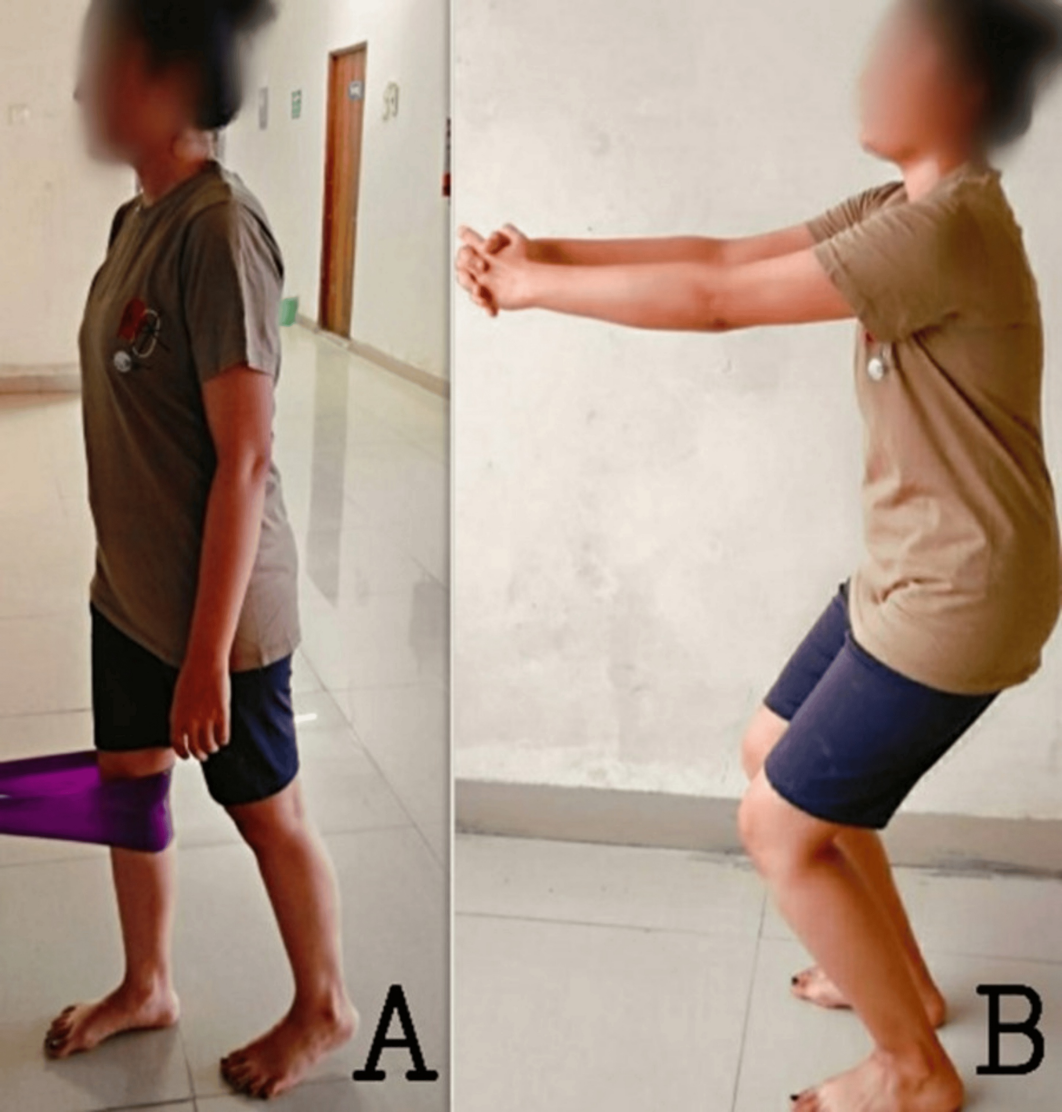
Closed kinetic chain exercises for the knee to improve function and to reduce pain: a) terminal knee extension with a theraband; b) squats

Closed kinetic chain exercises for the knee to improve function and to reduce pain are shown in Figure 1. 

**Figure 2:**
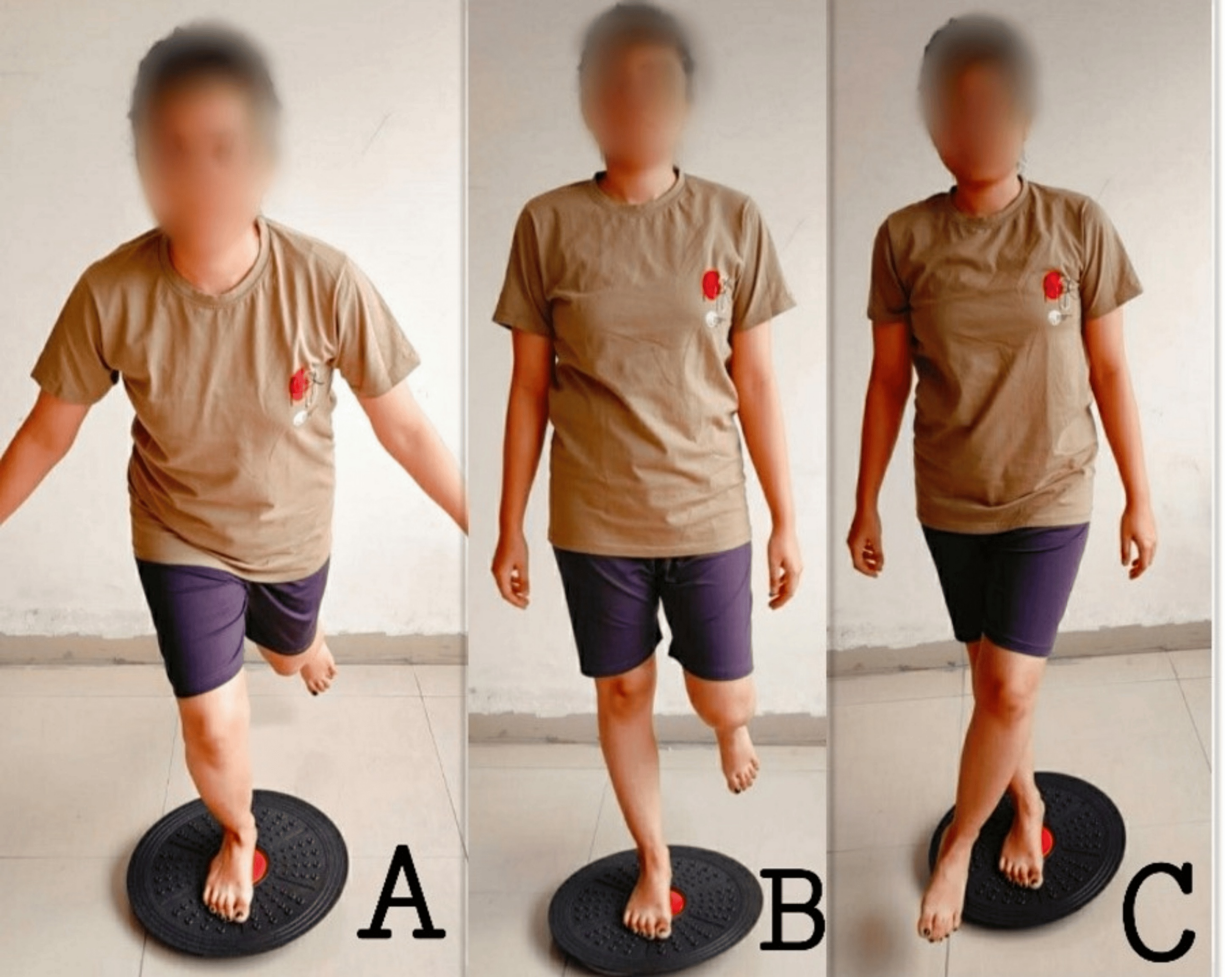
Proprioceptive exercises using a wobble board to enhance neuromuscular control and joint awareness: a) unilateral knee flexion; b) single-leg stance; c) crossed leg sway.

Proprioceptive exercises using a wobble board to enhance neuromuscular control and joint awareness are shown in Figure 2. 

Outcome measures 

Pre- and post-physiotherapy intervention outcomes are mentioned in Table 5, which show a significant improvement.

**Table 5:** Pre- and post-physiotherapy intervention outcome measures NPRS: Numerical Pain Rating Scale; KOOS: Knee Injury and Osteoarthritis Outcome Score

Sr. no.	Outcome measures	Pre-treatment	Post-treatment
1	NPRS on running	7/10	0/10
	NPRS at rest	3/10	0/10
2	KOOS	55%	100%

## Discussion

PFPS presents a multifaceted challenge in both diagnosis and treatment due to its diverse etiology and varied clinical presentations [16,17]. Despite being a prevalent condition, its complexity is underscored by these factors [18]. Previous research has highlighted several challenges associated with managing PFPS, including high recurrence rates and the limited efficacy of traditional treatments [19,20]. In addition, disruptions to position sense receptors caused by various knee injuries, including PFPS, have been shown to impact knee joint proprioception [21]. This discussion aims to explore key aspects highlighted in the case presentation and intervention strategies shedding light on the complexities of managing PFPS, particularly in a recreational long-distance runner. The case presentation underscores the typical clinical manifestations of PFPS, characterized by bilateral retropatellar pain exacerbated by weight-bearing activities, such as running, squatting, and stair climbing. The patient's symptoms, which had developed gradually over six months and were not associated with a specific traumatic event, align with the insidious onset commonly observed in PFPS cases. Moreover, the presence of decreased range of motion, strength, function, and proprioception further emphasizes the multifactorial nature of PFPS, implicating abnormalities in both biomechanical and neuromuscular domains.

The multifactorial approach included strengthening exercises focusing on hip adductors and lateral rotators, which play a crucial role in stabilizing the patellofemoral joint during dynamic movements like running and squatting. In addition, closed kinetic chain exercises such as terminal knee extensions and step-ups were employed to improve knee function and proprioception, essential for restoring optimal movement patterns and reducing joint stress. Flexibility exercises targeting tight muscle groups like the hamstrings, gastrocnemius, and iliotibial band were incorporated to enhance joint mobility and alleviate muscular imbalances contributing to PFPS symptoms. Proprioceptive training using a wobble board aimed to enhance neuromuscular control and joint awareness, facilitating better dynamic stability during weight-bearing activities. McConnell patellar taping was utilized to provide external support and optimize patellar alignment, thereby reducing pain and improving biomechanical efficiency. These interventions were systematically applied over a structured treatment period, emphasizing progressive overload and adaptation to ensure sustained improvements.

The outcomes of the intervention demonstrated significant symptomatic relief and functional enhancement. The patient reported reduced pain levels during activities that previously exacerbated symptoms, such as running and descending stairs. Functional assessments, including single-leg squats and step-down tests, showed improved stability and movement control. Objective measures, such as joint positional sense and manual muscle testing, indicated gains in proprioception and muscular strength, respectively, challenges in managing PFPS persist, including the potential for recurrence and variability in treatment response among individuals. Long-term follow-up and continued research are essential to evaluate the durability of treatment effects and refine therapeutic strategies. Future studies should explore novel interventions or combinations of therapies to optimize outcomes and address the complex pathophysiology of PFPS effectively.

## Conclusions

This case study illustrates the effective physiotherapy management of PFPS in a recreational runner. The holistic approach targeting strength, function, flexibility, proprioception, and pain management led to significant improvements in symptoms and functional outcomes. This underscores the importance of personalized, multifaceted treatments for PFPS and suggests avenues for future research to refine therapeutic strategies and enhance patient outcomes.

## References

[1] Pereira PM, Baptista JS, Conceição F, Duarte J, Ferraz J, Costa JT (2022). Patellofemoral pain syndrome risk associated with squats: a systematic review. Int J Environ Res Public Health.

[2] Kasitinon D, Li WX, Wang EX, Fredericson M (2021). Physical examination and patellofemoral pain syndrome: an updated review. Curr Rev Musculoskelet Med.

[3] Tramontano M, Pagnotta S, Lunghi C, Manzo C, Manzo F, Consolo S, Manzo V (2020). Assessment and management of somatic dysfunctions in patients with patellofemoral pain syndrome. J Am Osteopath Assoc.

[4] Fick CN, Jiménez-Silva R, Sheehan FT, Grant C (2022). Patellofemoral kinematics in patellofemoral pain syndrome: the influence of demographic factors. J Biomech.

[5] Reijnders L, Van de Groes SA (2020). The quality of life of patients with patellofemoral pain-a systematic review. Acta Orthop Belg.

[6] Emamvirdi M, Hosseinzadeh M, Letafatkar A, Thomas AC, Dos'Santos T, Smania N, Rossettini G (2023). Comparing kinematic asymmetry and lateral step-down test scores in healthy, chronic ankle instability, and patellofemoral pain syndrome female basketball players: a cross-sectional study. Sci Rep.

[7] da Silva Boitrago MV, de Mello NN, Barin FR, Júnior PL, de Souza Borges JH, Oliveira M (2021). Effects of proprioceptive exercises and strengthening on pain and functionality for patellofemoral pain syndrome in women: a randomized controlled trial. J Clin Orthop Trauma.

[8] Clifford AM, Dillon S, Hartigan K, O'Leary H, Constantinou M (2020). The effects of McConnell patellofemoral joint and tibial internal rotation limitation taping techniques in people with patellofemoral pain syndrome. Gait Posture.

[9] Yañez-Álvarez A, Bermúdez-Pulgarín B, Hernández-Sánchez S, Albornoz-Cabello M (2020). Effects of exercise combined with whole body vibration in patients with patellofemoral pain syndrome: a randomised-controlled clinical trial. BMC Musculoskelet Disord.

[10] Albornoz-Cabello M, Barrios-Quinta CJ, Barrios-Quinta AM, Escobio-Prieto I, Cardero-Durán ML, Espejo-Antunez L (2021). Effectiveness of tele-prescription of therapeutic physical exercise in patellofemoral pain syndrome during the COVID-19 pandemic. Int J Environ Res Public Health.

[11] Martinelli N, Bergamini AN, Burssens A, Toschi F, Kerkhoffs GM, Victor J, Sansone V (2022). Does the foot and ankle alignment impact the patellofemoral pain syndrome? a systematic review and meta-analysis. J Clin Med.

[12] Pollatos D, Chandolias K, Giordamni MA, Chalkia A, Trevlaki E (2021). Review of new data in physiotherapeutic approach to patellofemoral pain syndrome (PFPS). J Biosci Med.

[13] Callaghan MJ, Selfe J, McHenry A, Oldham JA (2008). Effects of patellar taping on knee joint proprioception in patients with patellofemoral pain syndrome. Man Ther.

[14] Coelho VK, Gomes BS, Lopes TJ, Corrêa LA, Telles GF, Nogueira LA (2021). Knee proprioceptive function and physical performance of patients with patellofemoral pain: a matched case-control study. Knee.

[15] Vikhe C, Ramteke S (2024). Effect of mindfulness training in adjunct to conventional physiotherapy on pain, function, and mindfulness attention awareness score in recreational long distance runners with patellofemoral pain syndrome: a randomized controlled trial protocol. F1000Research.

[16] Witvrouw E, Tiggelen DV, Thijs Y (2011). Intrinsic risk factors for patellofemoral pain syndrome: Implications for prevention and treatment. J Sci Med Sport.

[17] Waryasz GR, McDermott AY (2008). Patellofemoral pain syndrome (PFPS): a systematic review of anatomy and potential risk factors. Dyn Med.

[18] Smith BE, Selfe J, Thacker D (2018). Incidence and prevalence of patellofemoral pain: a systematic review and meta-analysis. PLoS One.

[19] Witvrouw E, Callaghan MJ, Stefanik JJ (2014). Patellofemoral pain: consensus statement from the 3rd International Patellofemoral Pain Research Retreat held in Vancouver, September 2013. Br J Sports Med.

[20] Stathopulu E, Baildam E (2003). Anterior knee pain: a long-term follow-up. Rheumatology (Oxford).

[21] Baker V, Bennell K, Stillman B, Cowan S, Crossley K (2002). Abnormal knee joint position sense in individuals with patellofemoral pain syndrome. J Orthop Res Off Publ Orthop Res Soc.

